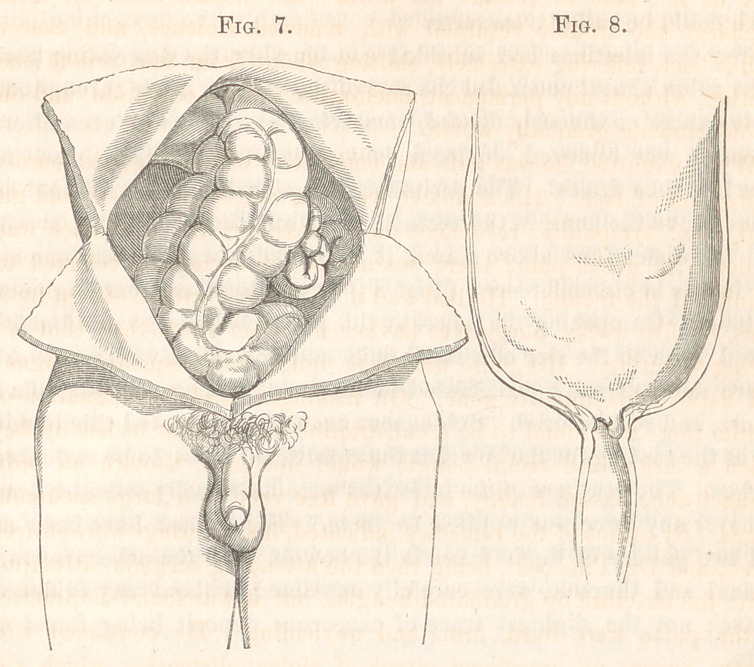# Proceedings of the Pathological Society of Philadelphia

**Published:** 1859-03

**Authors:** 


ART. IV.—*Proceedings of the Pathological Society of Philadelphia.*

WEDNESDAY EVENING, December 8th, 1858.

The President, DR. LA ROCHE, in the Chair.

*Wounds of the Heart.*—DR. DA COSTA exhibited a specimen of a wound of the heart, and read the history of another, for which he expressed his indebtedness to Dr. S. P. Brown.

CASE I.—A boy, aged eleven, while running about the fields with another boy shot himself. He was immediately conveyed to his home, not far distant, and lived, although the ball had entered directly over the heart and perforated the lung, for three hours after the accident. During this time he spoke repeatedly, and completely exonerated his companion from any blame.

On post-mortem examination the ball was found to have struck the right ventricle near the origin of the pulmonary artery, then to have glanced off to the artery, and from there to have touched several places in the left lung, passing out between the fifth and sixth ribs in the axilla. The pericardium was wounded, and contained some fluid blood. There was also some in the pleura.

CASE II.—An Irishman, Peter Conway, received two stabs in a fight, in which he endeavored to separate the combatants. It was not noticed at the time by the by-standers, although he was observed to have suddenly grown faint. He did not speak after being stabbed; he became exceedingly faint, was placed in bed, and remained quiet until his death, which occurred three hours after the injury. The wound in front was at the lower end of the ensiform cartilage, about an inch from the median line on the left side. The wound on the back was three inches from the spinous process, passing between the ninth and tenth ribs. The wounds had been inflicted with an instrument about five-eighths of an inch in breadth.

On post-mortem examination, six hours after death, it was found that the knife had passed through the pericardium, and into the lower part of the right ventricle, occasioning a horizontal cut, through which a scalpel-handle could be passed into the ventricle. The direction of the wound on the back was diagonal, and had probably cut the intercostal artery. It had passed through the pleura, but had not reached the lung. The pleura contained about six pounds of fluid blood, the pericardium none. All the other viscera were healthy.

DRS. GROSS, LA ROCHE, and HALL, each cited cases in which the heart had probably been wounded without immediate death resulting. In the case referred to by Dr. Hall, a large butcher's hook had entered deeply immediately over the region of the heart, and must from its direction have perforated the pericardium, and touched the heart. The patient recovered.

DR. ADDINELL HEWSON remarked that although the two specimens exhibited by Dr. Da Costa were exceedingly interesting, they were, as probably most of the members must be aware, far from being unique in their character. He had had occasion to investigate the subject very recently, and could cite many cases even more curious. Thus in Dupuytren's Essays could be found a number of such, including the famous case of the Duc de Berri, who was stabbed while leaving the opera by a man who stood behind him. The dagger was left sticking in the Duke's breast, and he himself drew it out, after he had been removed from the scene of the assault. Although the wound traversed the right auricle it did not prove fatal for several hours after its reception.

Thomas Bartholin mentions (Hist. Anat. Rar. Cent. 1, Hist. 77,) the case of a young man stabbed with a knife, in which the patient afterwards walked home some distance, and lived five days. Post-mortem examination revealed a small oblique wound of the right ventricle.

There are a number of cases on record in which persons have had the heart transfixed or penetrated with a stilet or darning-needle, and have entirely recovered. But perhaps the most extraordinary case on record is that reported to the Medical Association of the State of Alabama, by Dr. Chas. E. Lavender, of Selma, in 1850. It was in a student of medicine, aged nineteen years, who had been stabbed by a fellow-student; and from the account given by the Doctor, there can scarcely be any doubt that the knife penetrated the heart, (probably the right ventricle.) The patient lost over a gallon of blood, and recovered after an interval of twenty days' confinement. This extraordinary case is very fully quoted by Dr. Eve in his "Remarkable Cases in Surgery."

The direction of the lesion of the great central organ of the circulation in these and similar cases, was probably such as to separate, rather than divide the greatest part of the muscular fibres composing the wall at the wounded point, and hence probably the gaping was but slight, as has been pointed out by Dupuytren. This, no doubt, is also the reason why more cases have recovered from stabs, and penetrating wounds of the heart, from cutting instruments, than from injuries of a similar kind from gunshots. There are cases on record where shots have penetrated the cavities of the heart and yet death did not immediately follow. Dr. Randall, of Tennessee, has reported such a case, in a boy of fifteen years of age, who was accidentally shot by a fowling-piece, and survived the accident sixty-seven days. Three shot were found, after death, *lying loose* in the cavity of the ventricle, and two in the right auricle. The shot had entered the heart about one-third of the way from its base to its apex; the wounds made by them were at a little distance from each other; they had all cicatrized, but the spots were plainly to be seen. (See *American Journal of the Medical Sciences* for 1829.) But the most incomprehensible case of all is that mentioned by Dr. A. Christison in the *Edinburgh Monthly*. (Ranking's Abst., 1853.) In this case a musket-ball was found in the left ventricle, covered with lymph, without any traces of wound of the heart that could be discovered. The man, a soldier, had been shot some time previous by a musket-ball in the left shoulder. On dissection the course of the ball could not be traced among the textures of the shoulder, but could between the second and third ribs; it passed obliquely through a narrow canal with cartilaginous sides, and then through the costal pleura. A large abscess occupied the cavity of the pleura, except superiorly where there was air. The lung was very much condensed and pressed toward the heart; an opening in its pleural covering showed the continuation of the course of the ball, and this was traced onward as far as the root of the lung, where they failed to trace it farther. Dr. Christison thinks that the ball could only have found its way into the ventricle by one of the pulmonary veins.

*Incised Wounds of the Intestine.*—DR. LENOX HODGE presented a specimen of wound of the intestine.

H. W., a colored man, was brought to the Pennsylvania Hospital on the evening of Friday, November 26th, with an incised wound of the abdomen. The wound was about three-fourths of an inch long, and situated half way between the umbilicus and pubes, and about one and a half inches to the left of the median line. During the twenty-four hours that he survived, he complained of pain throughout his abdomen, and vomited constantly. On Saturday emphysema appeared, and extended as high as the nipple on the left side, and nearly as high on the right; passed down as far as Poupart's ligament, and distended the scrotum to its utmost.

The post-mortem examination showed the peritoneal cavity to be filled with fæcal fluid and gas, and the small intestines to be wounded in three places. These wounds of the intestines were situated in the ileum; the lowest was about six inches above the ileo-cæcal valve, the other two were close together and about six inches higher up. Each of them penetrated through all the coats of the intestines; was about half an inch in length, and perpendicular to the course of the canal. They beautifully show the effort that nature makes in such cases, to close the wounds, by bringing the edges together, and everting the mucous membrane.

In the course of a discussion of the various treatments pursued in intestinal wounds, DR. GROSS stated, that he invariably adhered to the practice of promptly returning the omentum, and sewing up the external wound, so as to prevent hernia, which he believed would be an inevitable consequence unless stitches were employed. The external wound will not unite without them.

DR. EDWARD HARTSHORNE referred to a case, published at length in the tenth volume of the *Medical Examiner*, in which, although no stitches had been used, a perfect union of the abdominal walls had taken place within ten days after the accident. The patient's death was caused by internal strangulation; a loop of the intestine was so bound down by bands, the result of the previous inflammation, as to have produced an obstruction of the passage. The wound was found, after death, to have entirely healed; the recti muscles were well united.

*Tubercular Meningitis; Concentric Hypertrophy of the Heart.*—DR. ALBERT H. SMITH presented specimens of tubercular meningitis, and of concentric cardiac hypertrophy.

William Anderson, aged forty-seven, colored, native of Philadelphia, stevedore, entered the hospital, on the 30th of November, 1858. He had always had excellent health until three weeks previous to his admission, when he was attacked with pains through his limbs and back, general malaise, loss of appetite, and constipation. These symptoms were soon followed by a chill, accompanied with a violent throbbing pain in the head, which continued without abatement until the patient applied for admission. Beyond this, nothing definite can be said of his history; he had no convulsions, nor at any time loss of consciousness or power, or impairment of the special senses.

When he presented himself, he was of full habit; not emaciated; he was sitting with his legs upon a settee, body bent forward, and head thrown slightly backward, which position, he mentioned, was the most comfortable he could assume; the expression of his face was one of extreme misery. Upon being questioned, he answered quite coherently, though with the short hurried manner of one in great pain. His pulse was full, though compressible, quite regular, sixty per minute; tongue thickly coated with white fur; mouth dry; respiration natural; skin dry, but not hot. He had abdominal tympanitis, with slight tenderness on pressure over the whole region; his bowels had been quite loose for three days. His pupils were contracted to a point, and insensible to light. Vision scarcely impaired; all other senses perfect. The most prominent symptom was the violent throbbing pain through the head, which was constant, and without remission. To use his own expression, "the misery in his head almost drove him crazy." He was greatly debilitated, having been carried to the hospital. There was no abnormal condition of either the heart or lungs apparent from physical signs.

He continued in this state, without any change, for four days, when his symptoms became more alarming. His pulse was more frequent, weaker, and smaller; skin cooler and moist. He grew very restless, with occasional maniacal jactitation, at times jumping out of bed, calling upon persons for help whom he imagined around him. His sensibility and consciousness diminished, and a partial delirium set in; though upon addressing him in a loud voice, he would give a coherent and rational answer. His head, previously drawn back voluntarily, because it afforded him some relief, was now rigidly fixed almost at a right angle to the normal axis of the neck; his pupils were still obstinately contracted; there was slight convergent strabismus. Involuntary urination occurred, but no action of the bowels except by injection. There was no apparent affection of the lungs or heart. These symptoms continued with increasing violence until the eighth of December, when he sank under the exhaustion, and died eight days after admission. There was no paralysis, nor muscular rigidity, except that mentioned of the neck.

*Autopsy.*—Encephalon: Dura matter greatly congested; arachnoid cavity containing about six ounces of clear serum; arachnoid inflamed, opake, and thickened, with slight deposit of miliary tubercle on the superior surface of the posterior lobes along each side of the longitudinal fissure; none that could be discovered at the base of the brain; pia mater violently injected over its whole surface; substance of brain firm and healthy in appearance; lateral ventricles containing some effusion, rather more than the normal amount; choroid plexuses congested. Thorax: Pleuræ free from effusion or adhesions; pulmonary surface studded with miliary tubercles; costal surface entirely free from them. The whole substance of both lungs was completely filled with the same deposit. The heart was small, about two-thirds the normal size; on opening it, the walls of the left ventricle were found to be concentrically hypertrophied, being, at the middle, ten lines in thickness, and reducing the cavity of the ventricle to the capacity of from six to eight fluidrachms; there was no other abnormal condition of this organ, nor of the great vessels. Abdomen: Peritoneum free from tubercular deposit; liver large and fatty; spleen filled with the miliary tubercles through its substance; none on its surface; all the other viscera perfectly healthy.

To be certain that the heart was not in a state of post-mortem contraction it was kept for several days, and attempts were made to dilate it with the finger, but it underwent no change.

---

WEDNESDAY EVENING, December 22d, 1858.

The President, DR. LA ROCHE, in the Chair.

*Tumor of the Breast.*—DR. S. W. GROSS presented a tumor removed by Professor Gross, from the left breast of a woman forty-three years of age, at the Clinic of the Jefferson Medical College. She is the mother of seven children, having been confined, for the last time, five years ago. Her menstrual functions are performed regularly. About seven years since, she first noticed a small, hard, inelastic lump in the upper part of the breast, which did not give rise to any inconvenience up to the middle of last summer, a period of six and a half years; it had increased to about one-half its present size, that of a large fist, when she began to feel sharp pains shooting through the tumor, which caused her a good deal of suffering. There was no enlargement of the subcutaneous veins, no lymphatic involvement, nor was the nipple retracted. The diagnosis was that of a fibrous tumor. The woman made a good recovery, the wound having united by the first intention. The structure of the tumor looked very much like that of the udder of a cow.

Dr. Packard, under the microscope, (magnified 472 diameters,) detected—

1. Portions of lactiferous tubes, lined with epithelium in some portions of their extent, and at others showing merely the tube of basement membrane.
2. Fibrous tissue.
3. Fibro-plastic cells, in not very great abundance.

*Aneurism of the Subclavian Artery, Injected with Perchloride of Iron.*—DR. FORBES, in exhibiting this specimen, remarked as follows:—

William Brunte, aged thirty-six, was a native of Germany, and a farmer by occupation. While working in hay with a pitchfork, on the twenty-eighth of August last, he felt something give way about his shoulder-joint on the right side. He had pain throughout the entire extremity of that side, which, after a few hours, localized itself under the middle of the collar-bone. A pulsatory tumor made its appearance over the middle of the clavicle, and on the twelfth of September he left the farm, and came to this city for counsel. His surgeon pronounced it to be an aneurism, and advised that no time should be lost in seeking further aid in view of an operation, as the tumor was now as large as a hen's egg, and increasing rapidly in size. Upon further consultation, it was concluded not to operate. The tumor grew rapidly, and ten days after his arrival in the city, he sought the advice of a German physician, who proposed to him the injecting of perchloride of iron into the tumor as his only hope. This operation was accordingly performed; three distinct openings were made near the summit of the tumor, but what quantity of the perchloride of iron was injected I could not ascertain; nor could I learn what amount of hemorrhage occurred when these openings were made. Ten days after the operation of injecting was performed, the surgeon ceased his attendance, and on the following day the patient sent for Dr. Freeman, of this city, who, after repeated solicitations, and after being well assured that the German physician had ceased to attend, concluded to visit him.

The tumor was found to be larger than an ostrich egg above the clavicle, the margins of the three openings made for the purpose of injecting the perchloride of iron, were sloughing; there was no pulsation in any of the vessels of the limb; still it was warm, œdematous, and entirely deprived of sensation. The patient was quite anæmic from the hemorrhages which had occurred since the German physician had ceased to visit him. The first hemorrhage appeared, from the man's description, to have been a general oozing from the opening farthest from the clavicle, and was stopped by the application of a cold cloth. The second hemorrhage occurred some six hours later, and was only stopped by the patient fainting. He was ordered an anodyne, which was to be kept up; a little stimulus, with broth, and other liquid nourishment; and the cold to be constantly applied. The following day the patient was more comfortable. Acetate of lead, acetate of morphia, and tincture of digitalis alternately during the day, were ordered, and the broth diet was continued with the local application of a cold cloth wrung out of lead water and laudanum.

The following day the aneurism had increased very much in size. The patient was disturbed by a constant cough, and his sputa were tinged with blood, and at times a good deal of pus. He had another hemorrhage during the night, was very anæmic, and evidently much weaker. He was most carefully watched; but bleeding more or less every day, he gradually sank. On the third of December I was asked to see him. The tumor I found extended from the angle of the inferior maxillary bone on the right side to the acromion process of the scapula, and from near the spinous processes of the lower cervical vertebræ posteriorly to beyond the inter-clavicular space anteriorly. The head was pushed over to one side, and the patient was breathing with great difficulty. The three orifices which were made for the injection of the perchloride of iron had all merged into one large orifice at the summit of the tumor, through which orifice the aneurismal clot was now emerging and its surface undergoing decomposition. He complained a good deal of cough and of a sensation of oppression at the summit of the thorax on the side of aneurism—the tumor evidently pressed against the lung, the phrenic and the pneumogastric nerves, and the trachea. In this critical condition, the breaking down of the clot so exposed to the external atmosphere would have caused death at any moment from excessive hemorrhage. The clot seemed to be pressed from below upward, not spasmodically, as if by the heart's action, but continuously and quite forcibly, thus causing it to protrude through the orifice at the top of the tumor, like a hernia, thereby preventing hemorrhage; yet there was at times a slight oozing of blood mingled with the pus of the breaking down clot. The whole tumor from base to summit was covered with small shreds of lint and thickly painted with collodion. Anodynes, digitalis, and a nourishing diet were recommended, and the patient was closely watched. He bled no more, but continued to sink, and died from exhaustion just thirteen weeks after he felt something give way in his right shoulder. Twenty-four hours after death an *autopsy* revealed the following condition of the parts:—

The tumor had collapsed but very little. An incision was made from above the hyoid bone to the pubic bone below. The innominate artery was traced up; the tumor was pressing upon its upper part, and likewise against the right carotid, compressing it against the lateral surface of the bodies of the lower cervical vertebræ, and thereby interfering materially with its functions, together with those of its two important neighbors, the pneumogastric nerve and internal jugular vein. The sac of the tumor was now opened, and the huge clot removed. The sac consisted only of the external coat of the artery thickened a little by inflammation. After the clot was removed, a *lacerated* opening about a quarter of an inch in extent was found to exist in the internal and middle coats of the third or last division of the subclavian artery, viz., the part existing between the external border of the scalenus anticus muscle and the inferior border of the first rib. The man had evidently ruptured the internal and middle coats of this artery in the exertions of tossing hay, as was demonstrated by the jagged appearance of the wound. The blood had forced itself out, and dissected up the external cellular coat of the artery, had extended down the axillary artery a short distance, and had proceeded upward posterior to the scalenus anticus muscle, as far as the bifurcation of the innominate artery, embracing all the branches of the subclavian, so that the first part of the vertebral, the external mammary, and the superior intercostal, and the whole of the thyroid axis were denuded of their external coat by the blood dissecting it off. The blood had dissected up so much of the external coat of the internal mammary artery, as to have formed a tumor as large as a hen's egg, which pressed against the pleura, and the upper and anterior part of the lobe of the right lung, causing it to break down, and giving rise in part to the pectoral symptoms so prominent a few days before death. The blood had also followed up the branches of the thyroid axis toward the acromion and upward toward the thyroid gland, and had caused extensive pressure against the phrenic and pneumogastric nerves and the internal jugular vein, by its extraordinary dissection of the external coats.

There was no cephalic disease, and only in one spot anywhere was there the slightest sign of degeneration; this spot was in the aorta just at the giving off of the cœliac axis, where there existed a very small atheromatous deposit. The lungs were perfect except at the summit of the right lung, where the tumor had pressed. Had this man been seen in a few hours after the accident occurred, the artery could have been ligated a little above the lacerated wound, just at the margin of the scalenus muscle, with some hopes of success. The character of the lacerated wound was peculiar; it extended in the direction of the long axis of the vessel and not around it. It was on the anterior surface of the vessel, and was small in extent. The innominate artery was very short, and all the branches of the subclavian came off from its first portion before pasing beneath the scalenus anticus muscle. All these conditions of the patient indicate that had he been operated upon soon after the accident occurred, and before the extensive diffusion of blood, there would have been much reason to hope for the most favorable results.

*Cancerous Stricture of the Rectum.*—DR. ALBERT H. SMITH exhibited a cancerous stricture of the rectum:—

James Money, aged forty-one, laborer, native of Ireland, entered the Pennsylvania Hospital on the 18th of August, 1858, suffering with symptoms of intestinal obstruction. He stated that he had had an attack of intermittent fever ten years previously, since which time he had always been affected with constipation. Eighteen months ago he became so much worse, with distension of the bowels and inability to evacuate them without immoderate quantities of purgatives, that he applied for admission into the hospital, and was treated there for about two months, for constipation, at the end of which time he was so much relieved that he felt able to resume his work. Six weeks, however, after his discharge the trouble returned with renewed violence, and ever since he has been suffering extremely, shifting about from one practitioner to another, without any permanent amelioration of his condition, and on the whole growing gradually worse.

When admitted, he was pale and emaciated, with a haggard, care-worn expression of face. His abdomen was enormously distended, and gave a loud, clear, tympanitic resonance on percussion; the distension was attended with great pain; there was no special uneasiness referred to the vicinity of the rectum. He had had no fæcal evacuation, except by injections, for five weeks, purgatives having no other effect than that of producing vomiting. His pulse was weak, quick, and frequent; tongue pale and flabby; his appetite gone. On making a careful examination of his rectum there was found to be a constriction about eight inches up, beyond which a large-sized rectal bougie would not pass, though an ordinary stomach-tube passed readily, and gave free vent to a large accumulation of gas. By means of the remedies indicated, his sufferings were entirely relieved, and Dr. Pancoast, being then the attending surgeon, attempted to dilate the stricture by graduated bougies, but it was found impracticable, as the parts were hard, firm, and unyielding. He continued without change, except an occasional attack of violent distension, which yielded promptly to remedies, until about the first of October, when Dr. Norris, then on duty, considering it a case of a cancerous character, had it transferred to the medical ward. As I had just previously been moved into that ward, I was fortunate in being able to retain charge of the patient. At that time the stomach-tube could no longer be passed, a good-sized urethral bougie alone could enter. The attacks of distension now became more frequent, the abdomen enlarging enormously, and becoming tense, and hard. These attacks were accompanied with extreme pain and prostration; vomiting usually attended them, though never of stercoraceous matter. The emaciation increased, and the life of the patient was an agony to which there was seldom a remission. The size of the instrument that would pass the constricted position gradually diminished, until the small rat-tail bougies alone could be admitted; there was evidently a great tortuosity of the rectum, and no metallic instrument could be introduced.

On the 13th inst. he was attacked, as previously, with inability to defecate, great distension, and with intense agony, which very soon prostrated him. The usual remedies failed to give relief; the smallest-sized flexible catheter could not be made to pass the point of constriction. His strength gradually failed, anodynes in enormous doses produced no alleviation of his misery, and he sank under it the following morning.

FIG. 7.

FIG. 8.

*Autopsy.*—The appearance of the body was most striking; the head and both upper and lower extremities were wasted to the bone, while the trunk presented a resemblance to the old Greek amphora, the thorax being compressed to the size of a child's, and looking like a protuberance upon the spherical mass of the abdomen, which measured *more than five feet in circumference.* It had the firmness and resonance of a drum-head. An incision was made over the sternum, carried down into the abdominal parietes, where the skin literally cracked before the knife as it was lightly touched. When the incision had reached about an inch above the umbilicus there was a sudden explosive report, with the expulsion of fæcal matter and gas, and an immediate subsidence of the whole abdomen to about one-half its original dimensions. Examining for the cause of this I discovered that the colon lying across the abdomen had at this point given way, being no longer able to resist the distension from which the support of the abdominal parietes was now removed. Continuing the incision and exposing the whole contents, a most interesting condition was exhibited; the small intestines were in their normal position, though greatly enlarged; but the whole colon was loosened from its peritoneal attachment, the ascending taking a course diagonally to the left hypochondriac region, forming an acute angle, descending again over itself to the right iliac, from which it made a circular sweep into the pelvis to the rectum. There was no portion which could be called transverse.

After the intestines had subsided considerably, the descending portion of the colon almost concealed the rest of the viscera, being throughout its whole extent enormously dilated, measuring at one point, even after the distension was removed, *eighteen inches in circumference*, and in no place less than fifteen. The peritoneum appeared healthy. About eight inches above the anus, in the rectum, was a stricture of the gut, at which, from the dimensions above stated, it narrowed down to about one and a half inches in circumference; (Fig. 8;) it was hard and cartilaginous to the touch. On opening the bowel at this point the passage was found contracted down to the size of a small quill, and immediately below the orifice toward the anus was a pediculated tumor about as large as a pea, of a firm texture, and rose-colored. Subsequent examination proved this tumor, as well as the coatings of the bowel at the constricted point, to be undoubtedly scirrhus. The coatings of the intestines were universally softened, tearing whenever any force was applied to them. There must have been more than two gallons of liquid fæces in the bowels. All the other viscera, abdominal and thoracic, were carefully examined, without any evidence of disease; not the slightest trace of cancerous deposit being found elsewhere.

*Curiously Twisted Umbilical Cord.*—DR. ROBERT P. HARRIS presented a spirally formed umbilical cord obtained, the day previous to the meeting, from a healthy primipara, delivered of a well developed male fœtus. This cord formed a continuous helix from the placenta to the umbilicus of the fœtus, in which there were between thirty and forty turns, each measuring (before the pulsation ceased) about three-quarters of an inch in diameter. Notwithstanding this spiral arrangement, the cord, when measured in a direct line, was about the usual length of this conduit when normally formed, reaching, after the expulsion of the child, from the umbilicus, up around the neck of the fœtus, down over its chest and abdomen, and to the placenta which was still within the uterus and firmly attached. In making traction to deliver the placenta the spiral portion within the vagina was necessarily elongated, or straightened out. The external portion not so pulled, which was presented to the Society, constituted, when fresh, a helix composed of twenty-eight spiral turns.

DR. KELLER remarked that twists and knots in the cord present many points for study. Knots in the umbilical cord may be of a simple or of a complicated nature. They may be of long existence before the birth of the child, or they may form during parturition. In the latter case they are produced while the child passes through a noose which is situated on the os tincæ, or twisted about the child's body. Thus a knot more or less tightly tied is produced which can immediately be loosened without leaving the slightest impression by which to indicate that it has existed. Those knots originating from the movements of the fœtus are often highly important to its existence, because if such a knot be drawn very tight the circulation between the fœtus and the placenta is diminished, if not entirely suspended. Sometimes, however, we meet with such knots, and even with those of a more complicated nature, which, by the impressions in the Whartonian jelly, evidently prove that they have been a long time present. A case of that kind occurred in his practice, on the 10th of March, 1858, the patient being a native of Switzerland. After her delivery of a healthy and stout girl, the umbilical cord presented a double knot resembling the figure 8, with deep impressions eight lines from the child's navel.

The lady, whom he had attended in confinement several times before, exhibited this abnormity for the first time.

In the course of some further obstetric remarks, Dr. Keller mentioned that he had recently delivered a lady of twins in whom he had found a perfectly movable xyphoid cartilage.

*Report of the Committee on a Diseased Scapula.* (See Proceedings of November 24th.)—The committee appointed to examine the scapula removed from a patient who had undergone amputation at the shoulder-joint, would respectfully report, that they considered the fragment lodged at the upper border of the glenoid cavity to be the acromion process of the scapula broken off and displaced downward.

This conclusion was arrived at accurately by comparative measurements, as follows :—

| | NORMAL BONE. Inches. | SPECIMEN. Inches. |
|---|---|---|
| Length of spine of scapula . . . | $5\frac{6}{8}$ | $4\frac{1}{2}$ |
| Acromion process . . . . . . | 2 | $1\frac{1}{4}$ |

At the acromio-clavicular junction there were all the evidences of an articulation, such as a sac containing synovial fluid.

As to the change of form in the glenoid cavity, the committee are led to the conclusion that it was subsequent to the amputation, the statement of the surgeon amputating, that the glenoid cavity was healthy at that time, inclining them to this view.

HALL, HEWSON, PACKARD.

WEDNESDAY EVENING, January 12th, 1859.

The President, DR. LA ROCHE, in the Chair.

*Pericarditis.*—DR. GROSS exhibited a specimen of extensive pericarditis, affecting the whole of the cardiac portion of the pericardium. The masses of lymph hung on the membrane like delicate villi; some almost half an inch in length and an eighth of an inch in breadth. The left ventricle was slightly hypertrophied. No history of the case could be obtained.

*Hypertrophy of the Muscular Coat of the Bladder; Occlusion of the Prepuce and Inflammation of the Kidneys and Ureters.*—DR. GROSS exhibited a bladder, the muscular coat of which was hypertrophied:—

Thomas Snellard, twenty-one years of age, a native of Philadelphia, had been affected with incontinence of urine for the last fourteen years, and had not, during all that period, ever passed a continuous stream, the urine constantly dribbling away. This was attended with a dull, heavy pain in the pelvis, and a sharp lancinating pain in the lumbar region, which was most severe over the right kidney. Six years ago he received a wound in the sole of the right foot from a nail, and within a period of three years fourteen pieces of bone were discharged, leaving the foot very much contorted and deformed. During the past summer he was attacked with diarrhœa, but it was only five weeks previous to his death that he was confined to his bed, during which time his bowels were almost constantly relaxed, and he became extremely emaciated. His general health was always delicate; several of his aunts and uncles had died of consumption. He would never permit an examination of his bladder or penis, so that the cause of the vesical trouble was unknown to his attendant, Dr. McQuean. The cause of death was extreme exhaustion produced by the diarrhœa. The autopsy was made on the third of January, twenty hours after death, the body being much emaciated.

The muscular coat of the bladder was hypertrophied, the organ being a beautiful specimen of what is known as the columniform bladder. The ureters were enlarged and their mucous lining was the seat of a fine capilliform injection, and in some places of softening. The right kidney was enlarged, and contained about two ounces of pus, its fibrous investment being in some places opake and firmly adherent to the organ. The left kidney was somewhat smaller than natural. In searching for the cause of these effects it was found that the prepuce was adherent to the glans-penis and that a very small opening was all that permitted the escape of urine. Just behind this a large pouch had formed capable of containing a drachm of urine. This state of things, which could have been easily remedied, had never been suspected during life.

*Hernial Dilatation of Bladder; Dilatation of Ureters from Stricture of Urethra.*—DR. LENOX HODGE exhibited a hernial dilatation of the bladder:—

J. McC., aged sixty-four years, had had stricture of the urethra for more than twenty years. He had an almost constant desire to urinate, could pass but a few drops at a time, and was in the habit of making violent straining efforts, so much so as to cause prolapsus of the rectum, and extravasation of urine into the cellular tissue of the scrotum. He died in the Pennsylvania Hospital on the twenty-sixth of December.

Upon making the post-mortem examination we found a dilatable stricture of the urethra, extending from just behind the glans backward about eighteen lines, and a cartilaginous stricture about six lines long at the membranous portion, where ulcerations through the walls of the urethra were discovered. The muscular walls of the bladder were excessively hypertrophied, and the cavity extremely small; at the upper and posterior part of its right side was a hernial dilatation equal in size to that of the bladder itself; the walls of this dilatation were very thin, and consisted simply of mucous membrane, and thickened peritoneal covering. Each of the ureters was dilated to the size of the urethra. The kidneys were healthy. There was an osseous deposit about six lines in diameter in the spleen, but none in the heart or arteries.

*Meningitis in a Patient who had had Delirium Tremens.*—DR. LENOX HODGE presented a specimen of arachnitis. The brain was deeply injected throughout; the arachnoid was thickened and opake; its cavity contained about six fluidounces of serum, while the sub-arachnoid space was nearly or entirely empty.

W. T., from whose body this brain was taken, was admitted into the hospital on the fifth of January, for a fracture of fibula. Being an habitual drunkard, the symptoms of delirium tremens soon appeared, became complicated with those of meningitis, and he died on the evening of the eleventh.

DR. STILLÉ inquired whether there had been any symptom indicatory of the commencement of the inflammatory disease, and also whether the delirium was positively that of mania a potu. Had the patient the hallucinations peculiar to delirium tremens?

DR. HODGE replied that the patient was known to have been a hard drinker, and was said never to have consumed less than a pint daily. He had had hallucinations from the first, although he evinced no fear, and spoke of never having had delirium tremens.

DR. STILLÉ felt by no means satisfied that the man had had delirium tremens. Persons are often supposed to have delirium tremens who have in reality inflammation of the brain. Men are admitted into a hospital in whom after accidents, or otherwise, delirium is a prominent symptom. They come from a class of population who all drink more or less, and this fact is deemed sufficient to make out a diagnosis of delirium tremens. Many also are supposed to be insane, who are in reality laboring under meningitis. It becomes thus of the greatest practical importance to distinguish the delirium of inflammation of the brain from that produced by other causes.

DR. GROSS thought that delirium tremens might give rise to inflammation of the meninges. It did not seem to him of such great importance, as regards the treatment, to diagnose an inter-current meningitis. Depletives could in neither case be employed; anodynes would be the best remedies.

DR. HEWSON agreed with Dr. Stillé as to the overrating of the occurrence of delirium tremens in hospitals. He referred to the fact that meningitis was not a rare cause of death in the hospitals of Paris, while it is well known that delirium tremens is an affection there not often met with.

DR. PACKARD, alluding to the post-mortem appearances in delirium tremens, said that two cases occurred when he was resident in the hospital, in which congestion supervened with a fatal result; at least, this condition was detected by post-mortem examination in one of them, and the other case was remarkably similar. Both were in veteran drinkers; both had lasted somewhat over a week; in both there was great depression of spirits and dread of death, and extreme irritability of stomach. In both the symptoms were ameliorating, when suddenly violent convulsions, with screaming and frightful delusions came on, and proved fatal, in one case in twenty minutes, and in the other in four hours.

An autopsy upon the former showed congestion of the dura mater, pons varolii and choroid plexuses, and a good deal of effusion beneath the arachnoid. No important phenomena were noticed in the other viscera, except patches of inflammation on the inner surface of the stomach.

*Strangulated Hernia; Omentum Hypertrophied and Mistaken for a Diseased Testicle*—DR. ADDINELL HEWSON exhibited a portion of omentum which he had had occasion to remove in an operation for strangulated hernia about ten days previously. The specimen was somewhat curious in itself, and the circumstances attending the case made it of peculiar interest. The patient from whom it was removed was a German, aged about thirty-eight years, who had been the subject of a scrotal tumor for over two years. When this tumor first made its appearance, the patient's family physician supposed it to be an omental hernia. Subsequently the presence of a portion of intestine was recognized as composing a part of the tumor. This became strangulated, and two surgeons saw the case, but failed by the taxis to effect its reduction. As, however, the symptoms were not then urgent, they postponed operating, and in their absence the intestine returned. This all happened about six months after the first appearance of the swelling in the scrotum. The scrotal tumor, however, remained, and had increased much in size since its first appearance. It was hard, nodular, irregular in form, although somewhat pear-shaped, and presented unmistakable signs of the existence of fluid in its interior.

The patient then consulted, but at different periods, two other surgeons, who gave their opinion that his case was one of hernia, complicated with diseased testicle, and he was tapped three times by two of the four surgeons who had then seen him, for the removal of the fluid contained in the scrotum. The quantity drawn at either of these operations never amounted to more than a drachm or two, and was clear and straw-colored. The patient wore a truss very constantly from the first appearance of the hernial protrusion of the intestine, but in spite of this the gut again became incarcerated on two different occasions, and on both these occasions after it had resisted all efforts at reduction with the taxis, it returned of its own accord. In the former of these attacks it was incarcerated twenty-four hours, and in the latter about seventy hours, but at neither time were the symptoms urgent; there was never any stercoraceous vomiting.

On the occasion on which Dr. Hewson operated, the symptoms of strangulation had lasted over seventy-five hours, and the indications for the operation were well marked. The scrotal tumor was then large, about four inches in diameter, pear-shaped, and smooth, having lost the irregular form which it had possessed, and on opening the sac, the large mass formerly supposed to be a tumor of the testicle, was found to be a portion of omentum which had undergone fatty hypertrophy, and was adherent at one point to the coverings of the testicle.

The edge of this portion of omentum was very nodular, and formed a complete ring around the intestine. Some of the masses of fat were here as large as the testicle. Dr. Hewson remarked that it could thus be seen how readily a mistake could be made under such circumstances, especially as the imperfect English of the patient could not aid much in supplying the facts or the history of the case so necessary for accuracy in the diagnosis.

*Fatty Liver.*—DR. HALL exhibited, for DR. HOYT, a fatty liver removed from the body of a man aged fifty-two, who had been subject to attacks of bronchitis and of jaundice. The bowels were habitually constipated, and the stools dark and offensive. Ten days prior to death there was incessant vomiting. On post-mortem examination, the lungs presented no evidence of tubercle, but exhibited evidences of having been affected with bronchitis. The liver was markedly fatty; the other viscera were healthy. The intestines and peritoneum were deeply dyed with bile.

---

WEDNESDAY EVENING, January 26th, 1859.

Vice-President, DR. STILLÉ, in the Chair.

*Fibrous Tumor on the Walls of the Abdomen.*—DR. GROSS exhibited a tumor, partly of a fibro-plastic, and partly of a colloid nature :—

E. H., eighteen years of age, first noticed, thirteen months ago, a movable, firm, inelastic tumor, about the size of a hen's egg, just underneath the skin of the abdomen to the left of the umbilicus. During the early part of last summer it had increased to the size of a large orange. It grew from above downward, was not accompanied with enlargement of the subcutaneous veins, and did not give rise to pain or any inconvenience. At the time of the operation the tumor was about nine inches in length, of a solid and inelastic feel, and slightly movable. The linea alba was curvilinear, having been pushed over to the right side. The general health of the patient had always been good. On the twelfth of January the tumor was removed at the Clinic of the Jefferson Medical College, chloroform having been administered. It was found to be very firmly adherent to the muscular structures of the abdomen, which were atrophied and spread over its external surface. Great care was required to detach it from the transverse fascia. The main arterial supply was at its lower portion, from an enlarged branch of the superficial epigastric, which required a ligature. Several points of the interrupted suture were applied to the opposed sides of the muscles, and to the integument. These, with adhesive strips, a compress and roller, constituted the dressings. At the present time the wound is nearly healed.

Examined by Dr. Packard, under a magnifying power of 472 diameters, there were seen—

1. Colloid masses of variable size, generally irregularly oval in shape; outline dark; contents very slightly granular, and showing bright oval spots like nuclei clustered together.

2. Exquisitely fine pale granular cells, nucleated, with double vesicular nucleoli; often elongated.

3. Coarser cells darker colored, with large nuclei, sometimes with many nuclei. These cells were arranged in masses, with fine fibrous tissue intermixed.

4. Very fine fibro-plastic cells, with large nuclei, and one or more nucleoli.

*Myeloid Tumors of the Abdomen, involving the Head of the Pancreas; Perforating Ulcer of the Stomach.*—DR. DARRACH presented, for Dr. Levizey, specimens of myeloid tumors, with Dr. Levizey's account of the case. The case from which the accompanying specimens were removed was a patient of Dr. Hiram Corson, of Montgomery County, who has furnished the following notes of the case. The abdominal distension had during life been recognized as caused by the pancreatic tumor.

George Cramer, aged twenty-three, who had always enjoyed good health, with the exception of an attack of intermittent fever about two years ago, began to be slightly jaundiced in June last, and about that time discovered a small tumor of the size of a hickory-nut in the right iliac region. The jaundice steadily increased, but he continued to work up to August, when the lump became irritated by the ends of the sheaves while he was feeding a thresher. His physician directed him to poultice the tumor, and in a few days plunged a lancet into it, supposing it to be an abscess. Poultices were continued two weeks longer, and occasionally the swelling was probed, yet there was never any discharge from it.

The patient still continued to work until October, when he was treated by another physician for two weeks, with mercurials for disease of the liver. Then he came under Dr. Corson's care, who made the following note of the case on November 9th, 1858 :—

He is deeply jaundiced ; has a tumor in the right iliac region as large as a big orange—lobulated and solid ; skin over the tumor discolored in spots, being red and purple, with the fine branches of blood-vessels ramifying over patches of it ; the cicatrix of the puncture is a little elevated, with a very fine skin over it, which seems as though it were not connected with the parts below ; the right side over the liver is larger than the left ; dullness a little higher than usual on this side of the thorax ; on a level with the umbilicus, and near to the lower margin of the liver, is a distinct, hard body, giving no resonance,. though there is a narrow line of resonance between that and the liver. The other tumor appears to be entirely external. He complains much of the left knee ; there is great pain at the inner condyle, with some swelling just above it ; the urine is very dark, and colors muslin a deep yellow. He is costive, with no appetite, and gets but little rest. The mercurials were continued, with opiates at night, and blisters over the internal tumor and region of the liver, and a cathartic pill occasionally. His stools are light-colored.

November 27th.—Stopped the mercurials a week ago on account of sickness and loss of appetite, which has since returned ; the bowels have been moved once or twice daily ; blisters have been well kept up, and there is a lessening of the fullness over the liver ; urine unchanged ; skin very yellow ; external tumor double the size and greatly mottled ; left knee still painful, and swollen on the inside. He takes but little nourishment, but has not vomited since he was first seen; sleeps well under one-quarter to one-half a grain of morphia; he cannot leave the bed; is much emaciated; has a full pulse of a 104 at all times.

December 5th.—Patient is better; skin is more natural; pulse, which has been 104 for weeks, is now only 96; tongue clean and natural; thirst for cold water very constant; with a good appetite. Ordered no medicine but morphia at night; bowels moved every day; and the stools are of a much darker color.

1859, January 1st.—Appetite voracious for the last two weeks; permitted him to eat as much and what he pleased; pulse 104; external tumor growing rapidly, and covered with fine, vermilion-colored branches of blood-vessels, in patches; the tumor in the abdomen can now be distinctly seen, elevating the parietes for about three inches.

January 16th.—Appetite still inordinate; complains of much pain in the knee, but sometimes has pain in the abdomen, and then the knee is easy; sleeps pretty well; bowels moved once or twice daily; is frightfully emaciated.

January 22d.—For the last two days he has been quite easy, but died at twelve o'clock to-day. About twenty hours before death he had inward spasms, with difficult breathing, and seemed to suffer very much.

Autopsy twenty-four hours after death. Body excessively emaciated; the external tumor in the iliac region was found to extend from a little above the anterior superior spinous process of the ilium to the pubes, and toward the middle of the abdomen about four inches across.

In dissecting up this tumor it was found to be resting upon the external oblique muscle, and adherent only by connective tissue to that structure. On making a section of this growth an appearance similar to that of soft marrow was presented, with here and there patches of a pinkish hue. Under the microscope this structure was found to consist of fat, with other cells, characteristic of myeloid tumor, (as described by Paget.) Some of these cells were shown, by means of acetic acid, to contain a nucleus, nearly filling the cell, and with one or more nucleoli.

The internal tumor, which had been recognized through the integuments, was found occupying a part of the umbilical and right hypochondriac regions. It was attached along its lower margin to the transverse colon, above and posteriorly to the duodenum; the latter was found very much thickened. This tumor also involved the head of the pancreas, and was adherent to the omentum and surrounding structures. Examined by the microscope it presented the same appearance as above mentioned. The right lobe of the liver was found enlarged, very much congested, and infiltrated with bile. The gall-bladder was distended to two or three times its natural size, and filled with bile; its duct being large enough to admit the index finger with ease. This distension was probably caused by the pressure of the pancreatic tumor.

Firmly attached to the lobus Spigelii was found a third tumor, about two inches in diameter, and of a much darker color than either of the others, but microscopically the same.

The consistence of the pancreas was denser than normal. Upon opening the stomach an ulcer, which had perforated all of its coats, was found upon its posterior wall, a little below the lesser curvature, and nearly midway between the cardiac and pyloric orifice. An enlarged gland upon the outer surface of the stomach, and near the ulcer, acting as a valve, probably prevented any escape of fluids into the cavity of the abdomen; the patient never having shown any symptoms of peritonitis, and no evidence of the same was discovered upon dissection.

The kidneys and spleen were very much congested, but presented no other abnormal appearance. The knee was not examined, but from the symptoms of the patient, it may fairly be inferred that the same disease existed in the inner condyle. Heart and lungs healthy. Brain not examined.

DR. DARRACH spoke of the views of Paget and Lebert, who compared myeloid tumors in their structure to the marrow of bone. Paget refers to a tendency shown by them to occupy the same seat as epulis. Dr. Darrach thought that in this case the tumors had formed previous to last June, and caused the jaundice by pressure. The disintegration of the liver-cells may perhaps have had something to do with the death.

DR. GROSS spoke of the equivocal position of myeloid tumors in nosology, and expressed a belief that they would be ultimately placed among encephaloid growths. The ulcer in the stomach, he thought, partook of the same character as the mass in the pancreas. He moved that the specimen be referred for examination to a committee.

*Double Pregnancy—one Tubal, the other Uterine.*—DR. PACKARD presented a specimen of tubal pregnancy, sent to Dr. Hodge in 1849, by Dr. Craghead, of Danville, Virginia:—

The woman was a negress, aged thirty-five. She had had symptoms of an ovarian tumor; these suddenly became aggravated by the occurrence of collapse, and she aborted, discharging a well-formed fœtus of somewhat over three months. Two days afterwards she died.*

At the post-mortem examination, "the whole abdominal cavity was found filled, anteriorly, with coagulated blood, and posteriorly with serum, which had proceeded from the rupture of some of the vessels of the left

* Case reported in Am. Journal for 1850.

Fallopian tube, now greatly enlarged, and converted into a membranous sac, containing a fœtus of the same size as the one delivered *per vias naturales.*"

*Intracapsular Fracture of the Neck of the Femur; Fracture of the Trochanter Minor.*—DR. LENOX HODGE exhibited an intracapsular fracture of the femur:—

H. F., aged seventy years, having fallen while going down the stairs of the Merchants' Hotel, fractured the neck of his femur, and was admitted into the Pennsylvania Hospital, on the 11th of December, 1858. He lived for five weeks and three days, and died from the effects of a diarrhœa on the 18th of January, 1859.

An autopsy showed the fracture of the neck of the femur to be within the capsule, except for a short distance at one point. The trochanter minor also had been fractured at its base. This last had united, and was surrounded with callus, though not perfectly consolidated; in the other case there had been no attempt at union. The specimen thus exhibits at once the difference in the action of nature when the fracture is intracapsular and when it is not.

*Inguinal Hernia; Death from Peritonitis.*—DR. JAMES HUTCHINSON, in presenting the specimens, said:—James Mackay, aged twenty-seven years, had been the subject of a reducible hernia in his left groin for a number of years. About the close of November, while engaged in lifting heavy barrels, it became strangulated, and after suffering for twenty-four hours he applied to a surgeon, who placed him under the influence of chloroform and succeeded in reducing it by taxis.

Ever since the man has suffered from severe pains in his abdomen, accompanied with vomiting and constipation. He entered the hospital six weeks after.

At the time of his admission into the hospital a small firm tumor occupied the inguinal canal, and some fullness was observable in the course of the cord below. His pulse was seventy-two, and even during the paroxysms of pain it never at any time exceeded ninety. The features wore a pinched expression. He vomited incessantly. His bowels were readily acted upon by enemata, which invariably afforded relief, and which brought away large quantities of fæcal matter, which was on one or two occasions ribbon-shaped.

During the past week his symptoms had decidedly improved. The vomiting had entirely ceased, and the pain was present only at rare intervals. Suddenly, on Sunday evening, he was seized with the signs of peritonitis, and death took place in a few hours.

An autopsy was made eight hours after death. The abdomen was somewhat distended with gas which had escaped into the cavity of the peritoneum. Upon cutting down upon the course of the cord, a small piece of omentum was found in the inguinal canal; it was, however, not at all strangulated. The abdomen, when opened, was found filled with fæcal matter, which had escaped from the jejunum. The omentum was united to the abdominal parietes, just outside of the internal ring, and bound down with it a portion of the small intestines, so diminishing its caliber that a large-size urethral bougie could hardly be passed through it. Just above the constricted portion was a small ulcer, by means of which the perforation had taken place.

DR. GROSS gave a further history of the case. He had succeeded in reducing the hernial tumor, although it had been strangulated for several days, by placing the patient under one grain of morphine and employing chloroform. The man was in good health for three weeks afterwards, when he commenced to complain of some abdominal tenderness, for which he was sent to the hospital. Dr. Gross further stated that since the introduction of anæsthetics he seldom used the knife, even when strangulation had existed for thirty hours.

## Figures and Tables

**Fig. 7. Fig. 8. f1:**